# Commentary: Antidepressants and diabetes risk: why are there discrepant findings from cohort studies based on patient records and those based on serial phenotyping?

**DOI:** 10.1093/ije/dyv186

**Published:** 2015-09-13

**Authors:** Mika Kivimäki, G David Batty

**Affiliations:** Department of Epidemiology and Public Health, University College London, London, UK

The potentially harmful effects of antidepressants, one of the most prescribed groups of drugs worldwide, have been the subject of some debate. In 2004, the US Food and Drug Administration issued a ‘black-box’ warning on antidepressants (so called because the advisory text on the package insert is required to be framed by a black border), after its own systematic review indicated that such therapy was associated with an elevated risk of suicidal thinking, feeling and behaviour in young people.^1^^,^^2^ In this issue of the *International Journal of Epidemiology*, Azevedo Da Silva and co-workers make a timely and important contribution to the evidence base for another controversial potential side effect: antidepressant medication might predispose individuals to type 2 diabetes.^3^

The safety of drugs is carefully tested in randomized controlled studies before approval, but most trials offer only a relatively short period of surveillance. When the side effects are rare and/or have an extended induction period, they may be missed in these otherwise well-conducted studies. Numerous chronic diseases, including diabetes, have been shown to develop over several years and may plausibly represent an unforeseen effect of antidepressant medication, not least because early studies suggested some antidepressants have a role in weight gain.

Data on disease diagnoses are routinely collected in many high-income countries, and these records allow an examination of the slowly developing effects of medication use that could not be captured in the initial trials. One such early study was a nested case-control analysis within the General Practice Research Database which contains electronic medical records of more than 6.4 million UK-based individuals registered with a primary care physician between 1990 and 2005. In the 15-year follow-up, people who had used antidepressants for 2 years or more experienced almost a doubling of later diabetes risk compared with non-users.^4^ Several other studies utilizing medical records or questionnaire data on physician-diagnosed diabetes reported similarly worrisome findings.^5–9^ As diabetes causes well-documented damage tovarious organs, including eyes, kidneys, heart and brain, and reduces life expectancy, findings suggesting a link between a commonly used medication and this disease predictably attracted worldwide media attention.

A major limitation in studies supporting the antidepressant use-diabetes association, however, was their reliance on diagnosed diabetes. If antidepressant use truly increases the risk of diabetes, an association would also be expected with undiagnosed disease as well as the metabolic changes that precede diabetes onset, such as elevated fasting glucose and impaired glucose tolerance. In the UK Whitehall II study, glucose levels have been assessed repeatedly over time in clinical examinations facilitating identification of undiagnosed diabetes to supplement extant data on diagnosed diabetes.^10^ In accordance with results from other studies,^5–9^ antidepressant users experienced about three times the risk of physician-diagnosed diabetes observed in non-users.^11^ Antidepressant use was not, however, associated with undiagnosed diabetes at any follow-up examination, nor was it associated with repeat measures of fasting or post-load glucose levels.^10^ The US National Health and Nutrition Examination Study (NHANES), another highly phenotyped study, closely replicated this pattern of results: a link between antidepressant use and diagnosed diabetes but no association with undiagnosed diabetes.^11^

Using data from DESIR, an unusually well-characterized population-based cohort study primarily established to examine the insulin resistance syndrome, Azevedo Da Silva and co-workers bring more evidence to bear on the antidepressant medication-diabetes controversy.^3^ The investigators examined changes over time in β-cell function (HOMA2-%B) and insulin sensitivity (HOMA2-%S), in addition to glucose levels, using repeat data over a 9-year follow-up in 4700 French adults.^3^ Capturing this wide array of biomarkers is useful because impaired β-cell function and reduced insulin sensitivity are among the key pathological changes leading to hyperglycaemia and type 2 diabetes. Again, antidepressant use was not associated with change in any of the biomarkers of diabetes metabolism. The findings from the DESIR study,^3^ in combination with others,^10^^,^^11^ therefore raise serious doubts about the potential causal role of antidepressant medication in the development of diabetes.

What could explain the apparent discrepancy between, on the one hand, medical record-based studies and, on the other, highly phenotyped, ‘bespoke’ cohort studies? As Azevedo Da Silva and others suggest, disease ascertainment bias is a possibility.^3^ Antidepressant use may increase the likelihood of being diagnosed with diabetes because people undergoing treatment for depression, owing to their more frequent contact with healthcare providers, are also more likely to be diagnosed with other ailments. This would have the effect of generating a spurious association between antidepressant use and diabetes when using electronic medical records only or relying on self-reports of diagnosed diseases only, but would not bias the association with undiagnosed diabetes or diabetes biomarkers.

Current research strategies emphasize ‘big data’ approaches, including linkage to electronic medical records, as a cost-effective means of minimizing random error and therefore producing precise (although not necessarily bias free) measures of effect. The new findings by Azevedo Da Silva and colleagues, allied to a series of similar investigations, illustrate that highly phenotyped cohort studies with long follow-up continue to have a crucial role if we are to obtain reliable evidence on disease aetiology.

## Funding

Medical Research Council (K013351). Funding for Open Access publication also comes from MRC (K013351).

**Conflict of interest**: None declared.
